# Factors influencing students’ happiness, vitality, and self-esteem

**DOI:** 10.3389/fpsyg.2024.1463459

**Published:** 2024-12-10

**Authors:** Daiva Majauskiene, Natalja Istomina, Dovile Valanciene, Ruta Dadeliene, Aurelija Sidlauskiene, Tomas Aukstikalnis, Ieva Egle Jamontaite, Emilija Strazdaite, Ramune Zilinskiene, Milda Gintiliene, Asta Sarkauskiene, Albertas Skurvydas

**Affiliations:** ^1^Department of Rehabilitation, Physical and Sports Medicine, Faculty of Medicine, Institute of Health Sciences, Vilnius University, Vilnius, Lithuania; ^2^Department of Nursing, Faculty of Medicine, Institute of Health Sciences, Vilnius University, Vilnius, Lithuania; ^3^Faculty of Law, Vilnius University, Vilnius, Lithuania; ^4^Department of Rehabilitation, Kauno Kolegija, Higher Education Institution, Kaunas, Lithuania; ^5^Department of Sports, Recreation and Tourism, Klaipeda University, Klaipeda, Lithuania

**Keywords:** happiness, life vitality, health and healthy lifestyles, emotional intelligence, logical thinking, moral decision, childhood negative experience, personality traits

## Abstract

**Introduction:**

The happiness and well-being of individuals are among the most important components of life. However, there remains a lack of evidence regarding the relationships between students’ happiness, vigor, and self-esteem on the one hand and various complex factors on the other hand.

**Methods:**

We conducted a cross-sectional study involving 397 students from various Lithuanian universities. We determined students’ happiness, self-esteem, vigor, healthy lifestyles, perceived stress, personality traits, academic achievements and motivation by using validated scales.

**Results and discussion:**

The study indicates that while happiness and self-esteem are not linked to healthy lifestyles, vigor is positively associated with moderate-to-vigorous physical activity (MVPA) and healthy eating, and negatively with body mass index (BMI). Happiness and vigor inversely relate to stress, and happiness and self-esteem inversely relate to depression. Vigor is positively related to extraversion, and self-esteem to neuroticism. Happiness, vigor, and self-esteem are not linked to academic achievements, but happiness relates to non-utilitarian decisions, and self-esteem to emotional intelligence and non-utilitarian decisions. Happiness, vigor, self-esteem were not linked to intrinsic motivation to study, but self-esteem was inversely related to amotivation and childhood violence. This study fills the research gap and deepens the understanding of what determines students’ happiness and vigor, and self-esteem.

## 1 Introduction

Research indicates that the happiness and well-being of individuals are among the most important components of life and are influenced by a multitude of interrelated factors, such as physical and mental health, positive emotions, engagement in specific activities, life aspirations, education, age, occupation, income, personality, etc. (Steptoe et al., 2015; Steptoe, 2019; Skurvydas et al., 2021; Skurvydas et al., 2022b; Terry et al., 2022b; Folk and Dunn, 2024; Skurvydas et al., 2024). Moreover, well-being is intertwined with competence, emotional stability, engagement, meaning, optimism, positive emotions, relationships, resilience, self-esteem, and vitality (Huppert and So, 2013; Yıldırım and Güler, 2021).

Ongoing research into the connection between well-being, happiness, and health reveals intriguing possibilities. This finding suggests that impaired happiness might not only result from poor health but also contribute to an increased risk of disease (Steptoe, 2019; Ong and Steptoe, 2020). The mechanisms linking happiness to health involve lifestyle factors such as physical activity and dietary choices, as well as complex biological processes, including neuroendocrine, inflammatory, and metabolic pathways (Steptoe, 2019). Research indicates that higher levels of physical activity correlate with increased well-being, better quality of life, and reduced symptoms of depression and anxiety across various age groups (Steptoe et al., 2015; Steptoe, 2019; Bull et al., 2020; Čekanauskaitė et al., 2020; Skurvydas et al., 2021; Skurvydas et al., 2024).

Research has shown that happiness, vigor, and self-esteem are significantly associated with healthy lifestyles (Pate et al., 2015; Richards et al., 2015; Kristjánsson et al., 2010). For instance, it has been clearly established that vigor is positively related to moderate-to-vigorous physical activity (MVPA) and healthy food consumption but negatively related to body mass index (BMI) (Pate et al., 2015). Additionally, health behaviors are positively associated with greater self-esteem (Kristjánsson et al., 2010). Studies suggest that interventions promoting Mediterranean diets among adolescents can lead to improvements in self-esteem (Knox and Muros, 2017). Research consistently indicates that happiness, vigor, and self-esteem are closely linked to psychological well-being, with these factors being inversely related to perceived stress and depression (Segrin et al., 2007). It is important to note that psychological well-being is a multifaceted concept encompassing aspects such as positive relationships with others, autonomy, purpose in life, and personal growth, which also play a significant role (Ryff and Singer, 1996). Zhao et al. (2021) reported a direct interaction between self-esteem and academic achievement in school. O'Brien et al. (2006) maintain that most self-esteem researchers never claim that self-esteem is an important predictor of complex, but rather specific, behaviors such as academic performance.

Moreover, research indicates that personality traits play a significant role in determining vigor and self-esteem. Specifically, vigor and well-being are positively related to extraversion and negatively related to neuroticism (Gale et al., 2013; Anglim et al., 2020). Tabbodi et al. (2015) found a positive correlation between happiness and academic achievement. Other studies have shown that adverse childhood experiences also impact well-being, learning, and health (Brown et al., 2024). Duke et al. (2010) found that gender and the type of adverse childhood experience, including violence, were significant factors in the relationship between self-esteem and exposure to violence. Chen et al. (2022), with students, showed that psychological well-being and emotions play important roles in learning motivation. Research has shown that there are associations between utilitarian judgment and negative mood (depression) (Laing et al., 2022). Research has shown that self-esteem can have a significant impact on people’s relationships at school and work, mental health, physical health, and antisocial behavior (Orth and Robins, 2022).

Despite the studies mentioned above, there remains a lack of evidence regarding the relationships between students’ happiness, vigor, and self-esteem on the one hand and various complex factors on the other hand. These factors include healthy lifestyles (such as physical activity, sleep quality, eating habits, and healthy foods), physical health indicators (such as body mass index, BMI), mental health indicators (including depression, perceived stress, and impulsivity), academic achievements (in mathematics and native language), emotional intelligence, non-utilitarian decisions and logical thinking, personality traits, adverse childhood experiences, and intrinsic motivation and amotivation. The aim of our study was to fill this research gap and deepen the understanding of what determines students’ happiness, vigor, and self-esteem.

## 2 Materials and methods

### 2.1 Sample

In total, 397 first-and second-year bachelor’s students (308 females and 89 males, with average ages of 23.4 years for females and 21.8 years for males, *p* = 0.066) from various Lithuanian universities were randomly selected and included in this cross-sectional study.

### 2.2 Measures

#### 2.2.1 Sociodemographic and anthropometric data

Sociodemographic and anthropometric data were collected by the survey. Participants were asked to indicate their age, gender, family status, education, place of residence, and financial security. Height, weight, systolic blood pressure and waist circumference were also measured. We calculated the body mass index (BMI) indicator based on the height and weight values provided by the respondents.

#### 2.2.2 Academic achievements

To evaluate academic achievements, participants were required to report their final exam scores in mathematics, native, foreign, or biology.

#### 2.2.3 Physical activity (PA) and sedentary behavior (SB)

The physical activity (PA) and sedentary behavior (SB) of the participants were determined by the Danish Physical Activity Questionnaire (DPAQ). The DPAQ was adapted from the International Physical Activity Questionnaire (IPAQ) and differs from it by referring to PA in the past 24 h (for 7 consecutive days) instead of the past 7 days. The selected activities were listed on the PA scale at nine levels of physical exertion in metabolic equivalents (METs), ranging from sleep or inactivity (0.9 MET) to highly strenuous activities (> 6 METs). Each level (A = 0.9 MET, B = 1.0 MET, C = 1.5 METs, D = 2.0 METs, E = 3.0 METs, *F* = 4.0 METs, G = 5.0 METs, H = 6.0 METs, and I > 6 METs) was described using examples of specific activities of that particular MET level and a small drawing. The PA scale was constructed so that the number of minutes (15, 30, or 45) and hours (1–10) spent at each MET activity level on an average 24-h weekday could be filled out. This allowed for a calculation of the total MET time, representing 24 h of sleep, work, and leisure time on an average weekday (Aadahl and Jørgensen, 2003; Matthiessen et al., 2008).

#### 2.2.4 Sports habits

To determine sports habits, we asked the following question: ‘Are you currently exercising?’. The respondents had to indicate their sport habits on a scale from 1 to 4, where 1 is “I do not exercise”; 2 is “I exercise by myself”; 3 is “I exercise in a gym/health center”; and 4 is “I am in professional sports.”

#### 2.2.5 Sleep quality

The sleep quality of the participants was assessed by the Pittsburgh Sleep Quality Index (PSQI). The PSQI was developed by Buysse et al. (1989) and is a self-report assessment tool that evaluates sleep quality over a one-month period. A global score and seven component scores can be derived from the scale. The component scores are as follows: subjective sleep quality, sleep latency, sleep duration, sleep efficiency, sleep disturbances, use of sleeping medications and daytime dysfunction. Each component is scored on a scale from 0–3, with the total score ranging from 0–21, where a higher score indicates poorer sleep quality. A total PSQI score greater than 5 has been validated as being highly sensitive and specific in distinguishing good from poor sleepers across a number of populations (Carpenter and Andrykowski, 1998).

#### 2.2.6 Perceived stress

The 10-item Perceived Stress Scale (PSS-10) was used to measure the participants’ perceived stress levels (Cohen et al., 1983). The PSS-10 asks the participants to answer 10 questions about their feelings and thoughts in the past month on a five-point scale ranging from 0–4. Total scores ranged from 0 to 40. Higher scores indicate higher levels of perceived stress.

#### 2.2.7 Anxiety and depression

The Hospital Anxiety and Depression Scale (HADS) was used to assess symptoms of anxiety and depression. It consists of two parts: an anxiety questionnaire and a depression questionnaire. The anxiety part of the questionnaire contains 7 questions that most reflect the symptoms of generalized anxiety disorder, and the depression part of the questionnaire also includes 7 questions that focus more on anhedonia as the main symptom of depression (Snaith, 2003). Subjects are asked to answer questions based on their state over the past week. Answers are evaluated with points ranging from 0 to 3, and the anxiety and depression scores are summed and separated. The maximum number of points for each part is 21. When interpreting the results of each part, 0–7 points are considered normal, 8–10 points are considered possibly indicative of having a mood disorder, and 11–21 points are considered most likely to indicate having a mood disorder.

#### 2.2.8 Happiness

The Happiness index was assessed by using the question ‘Are you happy in life?’ with response options on a 10-point scale from 1 (“very unhappy”) to 10 (“very happy”).

#### 2.2.9 Emotional intelligence (EI)

Emotional intelligence (EI) was assessed using the Schutte Self-Report Emotional Intelligence Test (SSREIT) (Schutte et al., 1998). The SSREIT is a 33-item questionnaire divided into four subscales: perception of emotions (10 items), dealing with one’s own emotions (9 items), dealing with others’ emotions (8 items), and using emotions (5 items).

#### 2.2.10 Vigor

The vigor and depression of the participants were assessed by using the Lithuanian-language version of the Brunel Mood Scale (BRUMS-LTU), which consists of 24 items designed to assess tension, depression, anger, vigor, fatigue and confusion. In this case, we used only the Vigor subscale, whose items were energetic, active, lively and alert. Participants responded on a five-point Likert scale, where 0 = not at all, 1 = a little, 2 = moderately, 3 = quite a bit and 4 = extremely, with total possible subscale scores ranging from 0–16 (Terry et al., 2003; Terry et al., 2022a).

#### 2.2.11 Cognitive ability

Participants’ cognitive ability was measured by the Cognitive Reflection Test (CRT). The test items were developed by Frederick (2005). The test consists of three tasks in which the wrong answer is automatically selected after reading. The author states that it is possible to determine what kind of thinking system a person uses. The first system reflects intuitive decision-making, which is usually fast, automatic, requires minimal effort, is implicit, and is emotional. The second system, on the other hand, reflects thinking that is slower, more deliberate, requires more effort, is goal-oriented, and is more logical. The test consists of three questions: (1) A bat and a ball together cost $1.10. The bat costs $1.00 more than the ball. How much does the ball cost? _____ cents; (2) If it takes 5 machines 5 min to make 5 widgets, how long would it take 100 machines to make 100 widgets? _____ minutes; (3) In a lake, there is a patch of lily pads in a lake. Every day, the patch doubles in size. If it takes 48 days for the patch to cover the entire lake, how long would it take for the patch to cover half of the lake? _____ days. The measure is scored as the total number of correct answers. The CRT measures the cognitive process, i.e., the tendency to suppress an incorrect, intuitive response and arrive at a more conscious, correct response.

#### 2.2.12 High-conflict personal moral decision-making

To assess what moral decisions participants make (utilitarian or non-utilitarian) and to provoke conflict between controlled cognitive processes and automatic emotional process responses, high-conflict personal moral decision-making tasks were used. These were high-conflict personal moral dilemmas (Greene et al., 2004, 2008). Participants were asked to make decisions in 3 high-conflict personal dilemmas an answer of “appropriate” indicates a utilitarian response (e.g., to save more lives at the cost of one or to hurt somebody but save others), and an answer of “inappropriate” indicates a non-utilitarian response (e.g., not to save any lives because the respondent decides not to hurt anybody). For our research purposes, the total number of decisions and the percentage of utilitarian and non-utilitarian decisions were calculated.

#### 2.2.13 Academic motivation

Participants’ academic motivation was assessed by the Student Academic Motivation Scale (SAMS-21) developed by Kairys et al. (2017). The scale consists of 21 statements, two to four statements for each type of motivation described in Vallerand’s model, forming subscales, the scores of which are obtained by calculating the averages of the statements that make them up. The respondents rated their agreement with the statement on a 7-point Likert-type scale, where 1 – completely disagree and 7 – completely agree.

#### 2.2.14 Personality traits

Five personality traits (extraversion, agreeableness, conscientiousness, neuroticism, and openness) of participants were assessed by the Big Five Inventory (BFI). This self-report scale was developed by John et al. (1991). The 44-item English BFI was constructed to allow efficient and flexible assessment of the five personality dimensions when there is no need for more differentiated measurements of individual facets. Participants rate each BFI item on a 5-point scale ranging from 1 (disagree strongly) to 5 (agree strongly); scale scores are computed as the participant’s mean item response (i.e., adding all items scored on a scale and dividing by the number of items on the scale).

#### 2.2.15 Bullying and abuse experience in childhood

We asked the respondents, “Were you bullied as a child?” and “Did you experience a lot of physical and psychological violence as a child?.” The respondents indicated their bullying and abuse on a scale of 1 to 3, where 1 was “no,” 2 was “yes” and 3 was “I do not remember.” We asked “If you were bullied as a child, indicate how often?” and “If you experienced physical and psychological abuse as a child, indicate how often?.” The respondents indicated the frequency of their bullying and abuse on a scale of 1 to 4, where 1 was “Very rarely,” 2 was “Rarely,” 3 was “Often” and 4 was “Very often.”

#### 2.2.16 Self-esteem

Participants’ self-esteem was assessed by the Core Self-Evaluations Scale (CSES) developed by Judge et al. (2003). The questionnaire consists of 12 questions. This is a 12-item scale, and in this study, a 5-point Likert scale ranging from 1 (strongly disagree) to 5 (strongly agree) was used. The CSES measures a single factor: the communality of self-esteem, locus of control, generalized self-efficacy and emotional stability. Sample items included “I am confident I get the success I deserve in life” and “When I try, I generally succeed,” each of which was assessed by a 5-point Likert-type scale. A higher total score indicates higher core self-esteem.

#### 2.2.17 Eating habits

The frequency of consuming healthy and unhealthy food products was determined by questions prepared by using the nutrition section of the Finbalt Health Monitor questionnaire from an international adult study conducted in Lithuania in 1994 (Grabauskas et al., 2015). To understand eating habits, respondents were asked about the frequency of consumption of certain products (fish, red meat, processed meat, fresh vegetables, canned vegetables, fresh fruits, sweets, chocolate, sweetened drinks, sugar in coffee or tea, pasta, rice, porridge, poultry, fast food, boiled potatoes, fried potatoes, eggs, biscuits, cakes, other dairy products, yogurt) over the past week. Possible responses were “Never,” “1–2 days,” “3–5 days,” and “6–7 days.”

Eating breakfast and overeating. Eating breakfast was evaluated on a scale of one to three, where 1 is “no,” 2 is “sometimes,” and 3 is “yes.” Overeating was also assessed using a scale of one to three, where 1 is “no,” 2 is “rarely,” and 3 is “often.”

#### 2.2.18 Assessment of impulsivity

Impulsivity was assessed using the Barratt Impulsivity Scale version 11 (BIS-11) (Patton et al., 1995.) The BIS-11 is a 30-item questionnaire divided into three subscales: attentional impulsivity, scored with 8 items; motor impulsivity, scored with 11 items; and nonplanning impulsivity, scored with 11 items. The items are answered on a four-point scale ranging from 1 (rarely/never) to 4 (almost always/always). Total scores range from 30 to 120, with higher scores representing greater impulsivity.

### 2.3 Statistical analysis

All statistical analyses were conducted using SPSS methodology. We verified that all interval data followed a normal distribution using the Kolmogorov–Smirnov test. To examine the interaction between happiness, vigor, self-esteem and various influencing factors, we utilized linear regression analysis and extracted standardized coefficients (Beta) and *p*-values. Additionally, we calculated the Pearson partial correlation coefficient (considering gender) among happiness, vigor, and self-esteem. All results were considered statistically significant at *p* < 0.05. Furthermore, one-way ANOVA was used to examine how various factors are related to happiness, vigor, and self-esteem.

## 3 Results

Among our participants, 41.6% did not engage in any physical activity, while 37.8% engaged in physical exercises occasionally. Only 10.8% exercised at sports and fitness centers, and 9.8% were professional athletes. We found that 70.5% of our surveyed students had a normal BMI, 7.8% had a low BMI, 16.9% were overweight, and only 4.8% were obese (the average BMI was 22.8 ± 4.3 kg/m2). The average happiness score among the students was 7.27 (1.8), their self-esteem was 39.5 (8.3), and their vigor was 7.8 (3.7). Interestingly, 53.7% of our surveyed students rated their happiness as 8 or above. Students made non-utilitarian decisions in tasks involving a railway, a ship, and a virus in 73.8, 53.5, and 31% of the cases, respectively. The participants correctly solved an average of 55% of all logical problems. In childhood, 27% of our participants experienced bullying, while violence was reported by 54.9%. The average BIS impulsivity score was 65.7 (SD = 7.1). The internal motivation to learn score was 5.51 (SD = 1.02), whereas the amotivation score was 2.72 (SD = 0.95). The participants’ personality traits of extraversion, conscientiousness, agreeableness, neuroticism, and openness had average values of 31.1 (SD = 4.7), 40.5 (SD = 5.1), 43.1 (SD = 5.2), 36.9 (SD = 5.1), and 46.1 (SD = 5.1), respectively. The average grades for the final state examinations in mathematics and native language were 37.8 (SD = 12.5) and 62.8 (SD = 18.5), respectively.

**Figure 1:**
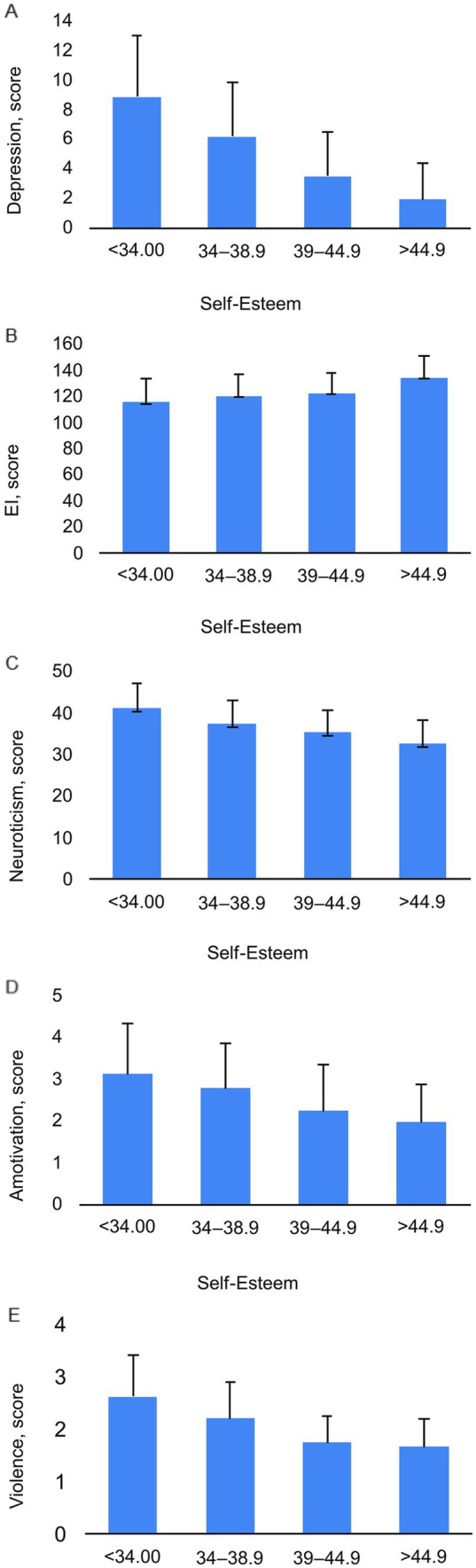
The Interaction of Self-Esteem with Depression **(A)**, Emotional Intelligence (EI) **(B)**, Neuroticism **(C)**, Amotivation **(D)**, and Violence **(E)**.

**Figure 2:**
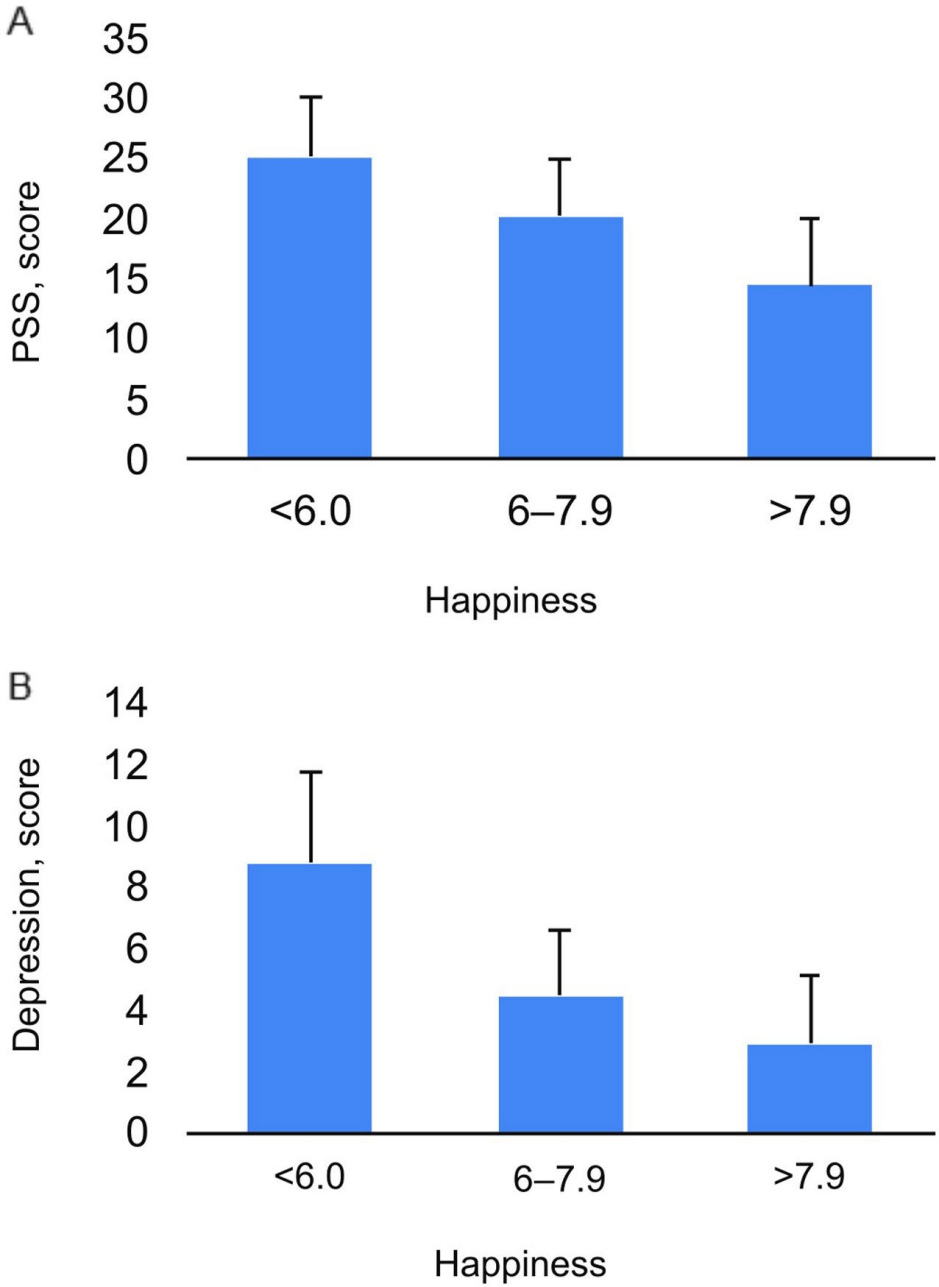
The Interaction of Happiness with Perceive Stress (PSS) **(A)** and Depression **(B)**.

**Figure 3:**
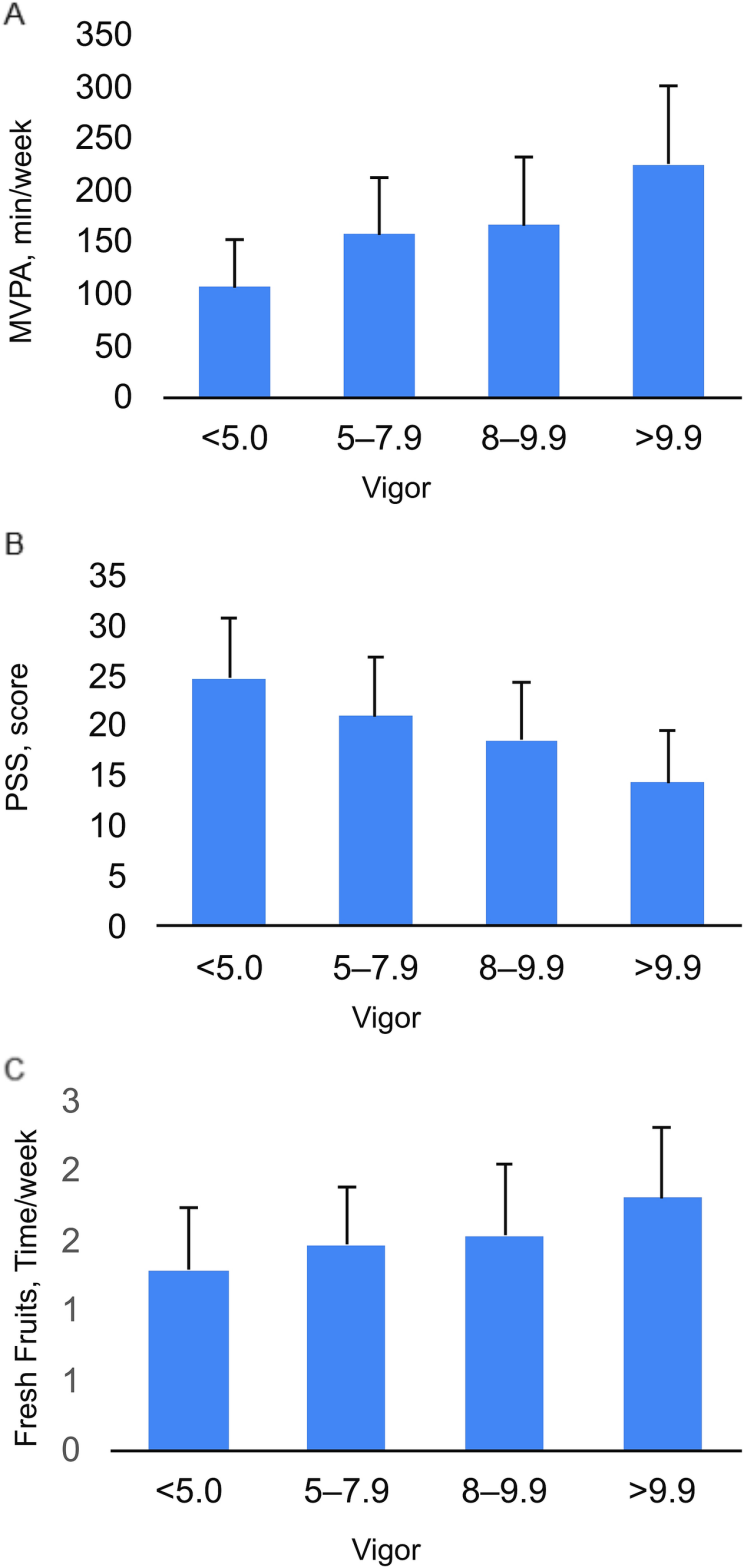
The Interaction of Vigor with Moderate-to-Vigorous Physical Activity **(A)**, Perceived Stress (PSS) **(B)** and Fresh Fruit Consumption **(C)**.

One-way ANOVA revealed that the effects of self-esteem on amotivation (*p* < 0.0001; ηp2 = 0.081), emotional intelligence (*p* < 0.0001; ηp2 = 0.16), depression (*p* < 0.0001; ηp2 = 0.375), personality trait neuroticism (*p* < 0.0001; ηp2 = 0.24), and violence (*p* < 0.0001; ηp2 = 0.11) were significant (Figure 1). We identified very significant interactions between happiness and the Perceived Stress Scale (PSS) (*p* < 0.0001; ηp2 = 0.28) and between happiness and depression (*p* < 0.0001; ηp2 = 0.27) (Figure 2). The impact of vigor on MVPA (moderate-to-vigorous physical activity) (*p* < 0.0001; ηp2 = 0.088), PSS (perceived stress scale) (*p* < 0.0001; ηp2 = 0.28), and fresh fruit consumption (*p* < 0.0001; ηp2 = 0.045) was significant (Figure 3).

The study results indicate that happiness and self-esteem are not significantly associated with physical activity, sleep quality, dietary habits (such as breakfast consumption and overeating), or BMI (Table 1). However, vigor was significantly positively related to moderate to vigorous physical activity (MVPA) and negatively related to BMI. Thus, the greater the BMI and the lower the MVPA are, the lower the students’ vigor. Happiness and vigor are strongly inversely related to perceived stress, while happiness and self-esteem are strongly inversely related to depression. Interestingly, none of these indicators (happiness, vigor, or self-esteem) are significantly influenced by impulsivity. Interestingly, neither happiness, vigor, nor self-esteem were significantly dependent on academic achievement (in mathematics or the native language) or the effectiveness of solving logical tasks. However, happiness was significantly directly related to non-utilitarian decisions, and self-esteem was related to emotional intelligence and non-utilitarian decisions.

**Table 1:** Dependence of happiness, vigor, and self-esteem (standardized beta coefficient and *p*-value) on healthy lifestyle, moods, psychological well-being, impulsivity, emotional intelligence, personality, childhood bullying and violence, and healthy foods.

	Happiness R^2^ = 0.478		Vigor R^2^ = 0.603		Self-esteem R^2^ = 0.74	
	*β*	*p*	*β*	*p*	*β*	*p*
Gender	−0.005	0.94	0.145	0.003	0.047	0.27
BMI	0.028	0.58	−0.118	0.007	−0.006	0.87
MVPA	−0.06	0.24	0.199	0.0001	0.002	0.86
Pittsburgh sleep scale	0.01	0.85	−0.048	0.31	−0.046	0.25
Breakfast eating	0.028	0.56	0.006	0.82	0.017	0.64
Overeating	−0.045	0.39	−0.077	0.086	−0.043	0.27
PSS	−0.328	0.0001	−0.235	0.001	−0.096	0.11
Depression	−0.234	0.001	−0.058	0.35	−0.21	0.0001
BIS	0.025	0.71	−0.093	0.089	−0.022	0.67
Mathematics	−0.025	0.65	0.09	0.86	0.051	0.25
Native language	0.048	0.38	−0.088	0.06	0.033	0.41
EI	0.083	0.21	0.034	0.57	0.188	0.0001
Non-utilitarian decision	0.124	0.031	−0.046	0.28	0.125	0.006
Logical task performance total	−0.032	0.51	0.047	0.58	0.012	0.74
Intrinsic motivation to learn	0.023	0.71	0.097	0.58	0.03	0.48
Amotivation	0.004	0.93	−0.036	0.51	−0.14	0.003
Extraversion	0.063	0.28	0.24	0.0001	−0.023	0.61
Conscientious	0.02	0.97	0.077	0.115	0.021	0.62
Agreeableness	0.089	0.17	−0.026	0.65	0.021	0.65
Neuroticism	−0.002	0.97	−0.034	0.57	−0.26	0.0001
Openness	−0.018	0.74	−0.02	0.68	0.033	0.41
Bullying	−0.034	0.54	−0.008	0.86	−0.033	0.42
Abuse	−0.052	0.34	0.064	0.19	−0.132	0.003
Red meat	0.022	0.64	0.027	0.53	−0.019	0.62
Processed meat	0.001	0.99	−0.12	0.01	0.02	0.65
Fresh vegetables	−0.002	0.96	0.028	0.58	0.003	0.88
Fresh fruits	−0.026	0.65	0.11	0.031	0.001	0.98
Sweetened drinks	−0.014	0.69	0.08	0.084	−0.013	0.75
Fast eating	−0.098	0.058	0.027	0.54	−0.039	0.31
Snacking	0.032	0.58	−0.054	0.22	−0.025	0.52

Happiness, vigor, and self-esteem were not dependent on intrinsic motivation to study. However, self-esteem was strongly inversely related to amotivation. Thus, the less motivated the students are, the lower their self-esteem. Interestingly, happiness was not significantly dependent on personality traits, while vigor was significantly positively related to extraversion, and self-esteem was significantly inversely related to neuroticism. Furthermore, the frequency of experienced violence in childhood was only inversely associated with self-esteem. Finally, our research data showed that only vigor was associated with healthy foods, was notably positively correlated with the consumption of fresh fruits and was inversely correlated with the consumption of processed meats.

A correlational data analysis revealed significant strong correlations between happiness and vigor (*r* = 0.49, *p* < 0.0001), between happiness and self-esteem (*r* = 0.53, *p* < 0.0001), and between vigor and self-esteem (*r* = 0.54, *p* < 0.0001).

## 4 Discussion

Our research revealed several interesting findings. Although happiness, vitality, and self-esteem are linked by many direct and indirect connections, our research data revealed several common and specific factors that influence them. We believe that these factors will provide a clearer understanding of what determines students’ happiness, vitality, and self-esteem.

First, the study results indicate that happiness and self-esteem are not significantly associated with healthy lifestyles; however, vigor is significantly positively related to moderate-to-vigorous physical activity (MVPA) and healthy food consumption and negatively related to body mass index (BMI). The fact that moods improve due to physical activity aligns clearly with our previous research (Skurvydas et al., 2021; Terry et al., 2022a; Terry et al., 2022b). For example, our recently published studies showed that moods particularly improve when individuals are physically active during their leisure time (Skurvydas et al., 2024).

Second, happiness, vigor, and self-esteem are significantly associated with psychological well-being: happiness and vigor are strongly inversely related to perceived stress (PSS), while happiness and self-esteem are strongly inversely related to depression. Research has shown that low self-esteem predicts vulnerability to depression (Franck and De Raedt, 2007). Numerous studies indicate that happiness and psychological well-being depend on various life factors, including physical and mental health, positive emotions, engagement in specific activities, life aspirations, education, age, occupation, income, personality, meaningful familial connections, etc. (Rodriguez-Ayllon et al., 2019; Steptoe, 2019; Bull et al., 2020; Skurvydas et al., 2021; Erickson et al., 2022; Skurvydas et al., 2022a; Skurvydas et al., 2022b; Skurvydas et al., 2022c; Folk and Dunn, 2024; Skurvydas et al., 2024). These factors are undoubtedly very interwoven, making it difficult to identify the underlying causes.

Third, vigor and self-esteem depend on personality traits; specifically, vigor is significantly positively related to extraversion, and self-esteem is negatively related to neuroticism. This finding aligns with the findings of other researchers that personality traits are linked to happiness, physical and psychological health, and spirituality (Ozer and Benet-Martínez, 2006). Additionally, other studies have shown that global satisfaction with social relationships is negatively associated with extraversion, neuroticism, and the ability to manage one’s emotions (Lopes et al., 2003). Additionally, our research data align with findings from other researchers showing that neuroticism and self-esteem are inversely related (Weidmann et al., 2017). Our research further showed that a more dominant extraversion personality is associated with greater vigor (vitality), while higher neuroticism is linked to lower self-esteem.

Fourth, happiness, vigor, and self-esteem are not significantly associated with academic achievements (in mathematics or the native language); however, happiness is directly related to non-utilitarian decisions, and self-esteem is related to emotional intelligence and non-utilitarian decisions. However, Zhao et al. (2021) found a direct interaction between self-esteem and academic achievement in school. Systematic analyses clearly show that physical activity, diet, and other behavioral interventions significantly improve cognition and school achievement, especially in mathematics, in obese or overweight children and adolescents (Martin et al., 2018). One of the latest systematic reviews indicated a small but positive association between adherence to all three recommendations of the 24-h movement guidelines (regular physical activity, reduced screen time, and optimal sleep duration) and enhanced academic achievement in children and adolescents (Bao et al., 2024). O'Brien et al. (2006) maintain that most self-esteem researchers have never claimed that self-esteem is an important predictor of complex but specific behaviors such as academic performance. Research has shown that there are associations between utilitarian judgment and negative mood (depression) (Laing et al., 2022).

Fifth, happiness, vigor, and self-esteem were not dependent on intrinsic motivation to study; however, self-esteem was strongly inversely related to amotivation. Studies conducted by Chen et al. (2022) with students showed that psychological well-being and emotions play important roles in learning motivation. Research shows that self-esteem can have a significant impact on people’s relationships at school and at work, mental health, physical health, and antisocial behavior (Orth and Robins, 2022). Zheng et al. (2020) clearly demonstrated that students with higher self-esteem tend to show improvements in their grades. Furthermore, achieving better grades and test scores fosters more positive self-perceptions. Research shows that students’ happiness is a good motivator for better learning (Haase et al., 2012).

Finally, only the relationship between self-esteem and the frequency of experienced violence in childhood was significant and inverse. This finding aligns with the conclusion from Shen’s (2009) study that there is a significant inverse relationship between self-esteem and childhood violence, especially concerning parental violence.

### 4.1 Limitations and directions for future research

The main limitations of our study are that we cannot establish mechanistic (causal) relationships between happiness, vigor, and self-esteem on the one hand and influencing factors (such as BMI, complexity of human emotions, physical activity, sleep quality, eating habits, etc.) on the other hand based on our research alone. Randomized controlled experiments are needed for this purpose.

## 5 Conclusion

The main strength of our study lies in how our empirical findings have broadened the understanding of the factors most strongly associated with students’ happiness, vigor, and self-esteem. Specifically, we explored their relationships with healthy lifestyles (physical activity, BMI, sleep quality, eating habits), mental health (stress, depression, impulsivity), personality traits, emotional intelligence, non-utilitarian decisions, logical problem-solving skills, experiences of bullying and childhood violence, academic achievements, and motivational components.

## Data Availability

The data analyzed in this study is subject to the following licenses/restrictions: data described in the manuscript and analytic code will be made available upon request pending application and approval. Requests to access these datasets should be directed to Daiva Majauskiene, daiva.maja@gmail.com.
